# Enhancing wind erosion risk assessment through remote sensing techniques

**DOI:** 10.1371/journal.pone.0308854

**Published:** 2024-10-31

**Authors:** Abdolhossein Boali, Narges Kariminejad, Mohsen Hosseinalizadeh

**Affiliations:** 1 Department of Arid Zone Management, Gorgan University of Agricultural Sciences and Natural Resources, Gorgan, Iran; 2 Department of Natural Resources and Environmental Engineering, College of Agriculture, Shiraz University, Shiraz, Iran; Universiti Kebangsaan Malaysia, MALAYSIA

## Abstract

Preventing wind erosion and dust storms has always been a major concern in arid and semi-arid areas because of their negative effects on the environment. This study aims to utilize remote sensing and machine learning techniques to model, monitor, and predict the risk of wind erosion in Northeast Iran. Through an examination of relevant studies, a comprehensive review was conducted, leading to the identification of eight remote sensing indicators that exhibited the highest correlation with field data. These indicators were subsequently employed to model the risk of wind erosion in the study area. Various methods including Random Forest (RF), Support Vector Machine (SVM), Gradient Boosting Machine (GBM), and Generalized Linear Models (GLM) were employed to carry out the modeling process. The final method utilized a weighted average of the model, and the SDM statistical package was used to combine different approaches to decrease uncertainty when modeling and monitoring wind erosion in the area. The modeling results indicated that in 2008, the RF model performed the best (AUC = 0.92, TSS = 0.82, and Kappa = 0.96), while in 2023, the GBM model showed superior performance (AUC = 0.95, TSS = 0.79, and Kappa = 0.95). Therefore, the utilization of an ensemble model emerged as an effective approach to reduce uncertainty during the modeling process. By employing the ensemble model, the outcomes obtained accurately depicted an elevated intensity of wind erosion in the northeastern regions of the study area by 2023. Furthermore, considering the climatic scenarios and projected land use changes, it is anticipated that wind erosion intensity will experience a 23% increase in the central and southern parts of the study area by 2038. By taking into account the reliable results of the ensemble model, which offers reduced uncertainty, it becomes feasible to implement effective planning, optimal management, and appropriate measures to mitigate the progression of wind erosion.

## 1. Introduction

One of the top environmental concerns today is the issue of wind erosion and the release of dust into the atmosphere. Wind erosion is a key factor contributing to desertification and land degradation in arid regions. This process limits soil fertility by transporting soil nutrients and fine particles. It poses a serious challenge to the sustainable production and management of agricultural lands, especially as it reduces surface soil depth [1]. While wind erosion may be less destructive than water erosion, its widespread and continuous impact makes it a crucial concern in dry areas [2]. Wind erosion and dust emission result in significant socio-economic losses and damages.

The Middle East accounts for about 25% of global dust generation [2], and in this region, wind erosion is a significant factor contributing to land degradation, particularly in arid and semi-arid areas like Iran [3]. Due to environmental factors such as limited rainfall and sparse vegetation, Iran’s semi-arid and dry areas are particularly susceptible to wind erosion. Additionally, rainfed lands in Iran, such as the dried wetlands and sandy deserts of Lut and Kavir, contribute to wind erosion and dust in Southwest Asia [4]. The economic impact of wind erosion in Iran is estimated to exceed 18 billion dollars annually [5]. In order to evaluate this form of erosion, estimate the amount of soil lost, and analyze variations in sensitivity among different regions, it is crucial to set up monitoring stations that are equipped with devices such as sediment traps and dust meters. These stations can be either stationary or portable. Nevertheless, the process of establishing and equipping a considerable number of stations, along with obtaining the necessary equipment, is both expensive and time-consuming. Furthermore, many existing models used to estimate wind erosion rely on coefficients that require additional research specific to each area or calibration based on local conditions [6, 7].

The primary challenge in utilizing these models is the need for extensive spatial and temporal data. Since wind erosion mainly takes place in arid areas, gathering extensive and long-term information is challenging [6]. Therefore, it is crucial to establish a comprehensive framework or system that addresses these challenges and facilitates effective wind erosion assessment and management [7]. Moreover, the main reason for predicting wind erosion in the future is that wind erosion significantly impacts environmental and ecological changes [2]. By predicting various factors such as wind speed, temperature, humidity, changes in land use, and other weather conditions, we can easily check the effects of each of them on wind erosion [8]. By using wind erosion prediction, we can plan and take appropriate measures to manage this erosion [9].

Overall, the novelty of this research lies in its utilization of advanced technologies, integration of remote sensing and machine learning, and the comprehensive approach to modeling, monitoring, and predicting the risk of wind erosion in Northeast Iran. The study’s findings provide valuable information for implementing measures to prevent the adverse impacts of wind erosion and promote sustainable development in the region. Evaluating current wind erosion models will be the starting point for this research. Next, each factor will be designated with a specific remote sensing index. By leveraging machine learning algorithms and integrating these remote sensing indicators, an ensemble model will be developed to accurately predict and observe wind erosion at different time scales. The main goal of this investigation is to provide useful guidance for land management, considering climate changes and expected alterations in land use to wind erosion.

## 2. Materials and methods

### 2.1 Study area

**Fig 1:**
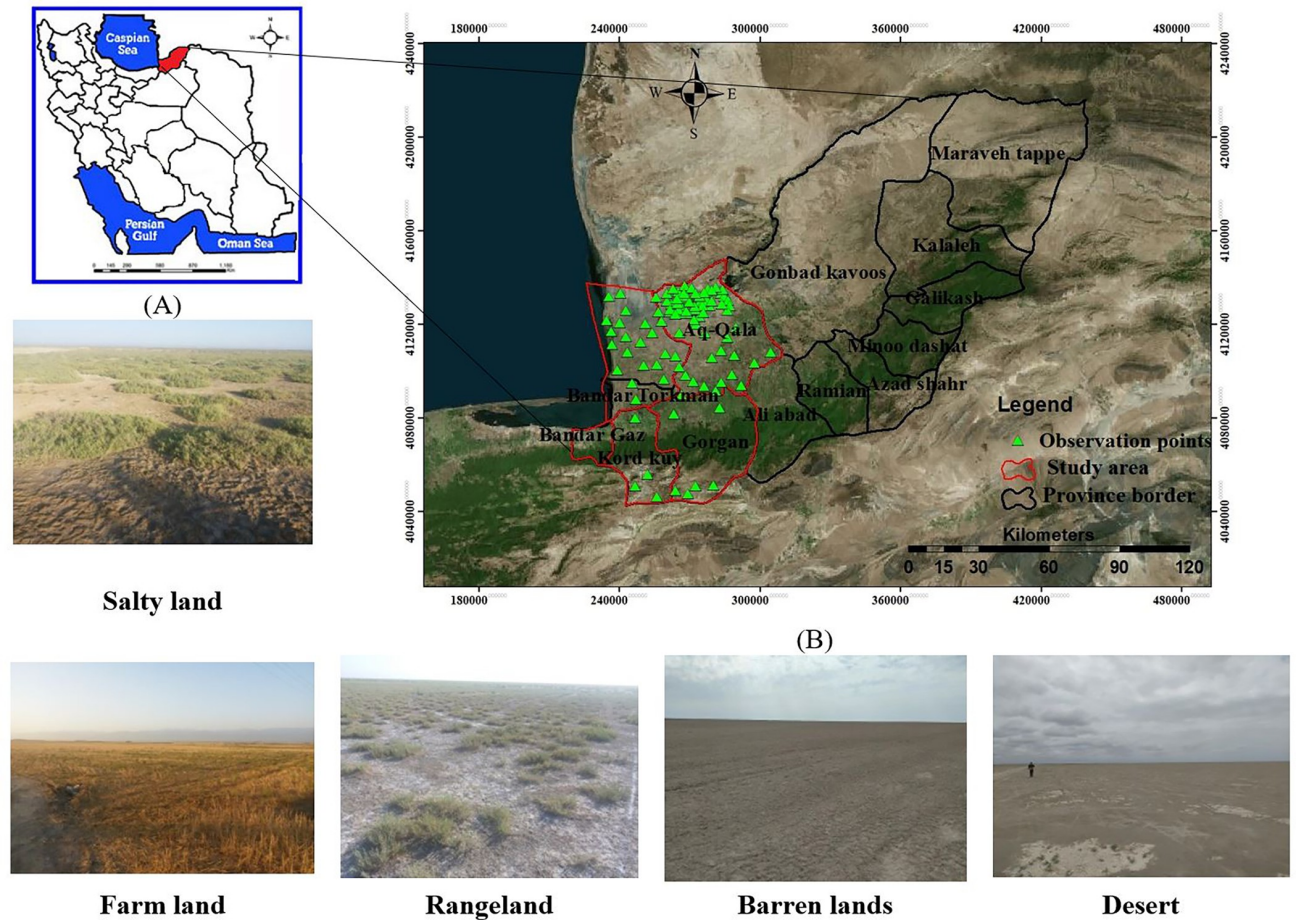
Geographical location of the study area in Iran (A), and Golestan province (B). All figures are generated in the ArcGIS 10.7.1 software (www.esri.com).

The study area is situated in the west of Golestan province, in the southeastern region of the Caspian Sea, the world’s largest lake, and the southwestern part of the Karagham desert in Turkmenistan. In the last ten years, this area has been facing challenges related to land degradation, erosion from water and wind, changes in land use, and the depletion of groundwater [10]. The area under investigation spans approximately 510100 hectares and is situated in the northeastern part of Iran, specifically in the northwestern region of Golestan province. This study focuses on the desert lands bordering Turkmenistan to the north, the coastal plain of the Caspian Sea to the west, the Alborz Mountain range to the south, and the city of Gonbad to the east (Fig 1). The highest elevation in the region reaches approximately 3088 meters above sea level in the southern part, while the lowest point is around -32 meters above sea level in the western part. Over 30 years (1994–2023), the average annual precipitation, evaporation, and temperature in the area were estimated to be 502 mm, 1338 mm, and 18.17°C, respectively.

### 2.2 Field data

Soil erosion in Golestan province is mainly caused by the presence of loess deposits and silty/silty-clay sediments, which are constantly changing. Field studies show that ongoing drought is a key factor in the rising levels of wind erosion and sand/dust storms. Extended periods of dry weather not only decrease vegetation cover but also lower soil moisture levels, making the soil more prone to wind erosion and adding to the frequency of sand and dust storms.

In July 2023, a total of 42 soil samples were gathered from the designated area to analyze the soil salinity and soil texture (S1 Data). To evaluate the vegetation, we established one-square-meter plots along a 100-meter path. This process was carried out systematically, with three paths allocated to each land use category and ten plots per path. Subsequently, the vegetation coverage within each plot was assessed by expert judgment and then extrapolated to represent the entire land use area. Furthermore, five meteorological stations were deployed to investigate various climatic factors, including rainfall, evaporation, transpiration, temperature, and wind speed. These stations collected data over a statistical period spanning 30 years, specifically from 1994 to 2023.

### 2.3 Google Earth Engine (GEE)

In 2010, the GEE computing platform was introduced by Google [11]. Over time, the GEE system has become increasingly popular among researchers as a valuable tool for accessing information and data in diverse fields. Using the GEE platform (https://earthengine.google.com) to analyze satellite images makes it possible to efficiently monitor wind erosion on a large scale and over a long period. Landsat Tier 1 surface reflectance images were selected for examination for this particular research. These images have undergone atmospheric and geometrical corrections to ensure their accuracy and reliability. Landsat images from 2008 to 2023 were employed to extract remote sensing indices and develop a wind erosion model.

### 2.4 Selection of remote sensing indicators to model wind erosion

The initial phase involves identifying the key factors that impact wind erosion in the area under investigation. This will be achieved by examining various remote sensing indicators associated with these factors. The remote sensing index showing the strongest correlation with ground surface data will be chosen for wind erosion prediction. The evaluation of correlations between indicators will rely on Pearson’s correlation coefficient, root mean square error, and detection coefficient. Moreover, factors like interpretability, spatial resolution, and temporal availability will also be taken into account when selecting the indicators.

### 2.5 Wind erosion modeling using machine learning algorithms

This research will employ machine learning techniques to analyze and track wind erosion. These approaches utilize data from remote sensing technologies and can serve as a robust and precise tool for modeling and assessing different occurrences [12, 13]. Various models have been used to evaluate dust and wind erosion events, including multivariate linear regression (MLR) [14], nearest neighbors (kNN) [15], artificial neural networks (ANN) [16], deep learning (DL) [17] and RF [17, 18]. While using a single method may not yield satisfactory results, combining these models can lead to more accurate outcomes. This study will utilize ensemble machine learning models from the sdm package to enhance the classification accuracy and better reflect reality [19]. Ultimately, the incorporation of ensemble techniques in our research is a strategic decision aimed at improving predictive accuracy and reliability, demonstrating the effectiveness of this approach in enhancing predictive modeling results.

To implement machine learning algorithms, the number of suitable training samples must be determined first. Based on the results of Lee et al. (2014) [8], there is no specific value for selecting the number of training samples. To reduce the spatial correlation, we selected 100 random samples based on pixels during field visits and visual interpretation of high-resolution Google Earth images of the region. These samples were used to implement machine learning algorithms. Specifically, 50 samples were chosen from areas where wind erosion has occurred, and another 50 samples were selected from areas where wind erosion has not occurred.

For the machine learning analysis, 70% of the samples were randomly assigned to the training group, while the remaining 30% were allocated to the experimental group. In this research, we employed four different machine learning methods: SVM, RF, GBM, and GLM. These methods have been previously studied and referenced by various researchers [20–22]. To justify the selection of machine learning methods in this research, four distinct algorithms were chosen, each serving a specific purpose. The RF was selected for its effectiveness in handling diverse datasets, mitigating overfitting, and identifying significant features. The SVM was employed due to its prowess in addressing complex problems and handling non-linear distributions, especially with large volumes of data. The GBM were utilized for their adaptability to noisy and complex datasets, along with their ability to manage random variables while retaining model interpretability. Lastly, the GLM was incorporated for its simplicity, interpretability, and applicability in scenarios where specific statistical assumptions are met. These selections collectively ensure a robust and comprehensive approach to data analysis in this study.

This research utilized the SDM R package [23] for analysis. Each machine learning approach was executed three times randomly to eliminate any potential bias from the sampling point distribution. The accuracy of the models was evaluated using three indicators, including the kappa coefficient, Receiver Operating Characteristic (ROC), and True Skill Statistic (TSS) related to the region, as suggested by Allouche et al. (2006) [24]. The kappa coefficient calculates the classification accuracy based on all the correctly and incorrectly classified pixels, and its range of changes varies from +1 to -1. The larger the kappa value, the better the classification method [25, 26]. The most ideal model based on the ROC curve index is the model that has the highest area under the curve and the values (AUC) vary from 0.5 to 1 [26] Excellent classes (0.9–1), very good (0.9–0.8), good (0.8–0.7), average (0.7–0.6), and poor (0.5) - 0.6) for the qualitative and quantitative correlation of the sub-curve level [27, 28]. The Tss statistic is defined as TSS = sensitivity specificity—1, where the sensitivity feature is related to the values that are correctly predicted, and the specificity feature is related to the values that are not correctly predicted [29]. The TSS classes should be considered poor (less than 0.2), moderate (0.2 to 0.6), and good (greater than 0.6) respectively [29]. To more accurately assess the performance of the models, we utilized the cross-validation method with k = 10. This involved dividing the data into 10 parts, with one part being used as test data and the remaining parts as training data in each iteration. This process was repeated 10 times, and the average performance metric (Accuracy) was calculated at the end. The significance of each statistical index was determined using R statistical software. The DeLong test was applied to the AUC index, the Z test to the Kappa index, and the McNemar test to the TSS index. Finally, we removed any model that performed worse and the remaining models were used to monitor wind erosion.

### 2.6 Monitoring and forecasting wind erosion

In some cases, relying solely on a single-variable model may result in errors because of a faulty or overly sensitive model, which can exacerbate the mistakes. In such scenarios, using an ensemble model can help alleviate errors by relying on a combination of different models. According to Bui et al. (2014) [30], by considering the results from a group of models, even if one model produces an error, the other models can still yield accurate outcomes. The Ensemble models have the advantage of leveraging the capabilities and strengths of different models while mitigating their weaknesses. By combining the strengths of various models, forecasting errors can be minimized, as highlighted by Lee et al. (2019) [15]. Moreover, the Ensemble models can identify more complex patterns by integrating information from multiple models, thereby enhancing the quality and accuracy of predictions, as mentioned by Wang et al. (2019) [31]. It’s important to note that this approach involves more than simply integrating models. Instead, it involves assessing the models’ performance, learning abilities, and adaptability to estimate their weights. The estimated weights are then used to calculate the ensemble model’s result through weighted averaging, as explained by Guo et al. (2020) [32].

This study utilized a combination of four machine learning models to address wind erosion monitoring. The SDM statistical package was employed to calculate the weighted average of the group model. Ensemble models, through their amalgamation of diverse decision-making processes, typically yield heightened accuracy compared to individual models, thus enhancing predictive performance [33]. Moreover, by consolidating predictions from multiple models, they often demonstrate lower variance, mitigating over fitting and enhancing generalization to unseen data. Additionally, the ensemble approach leverages the diversity of constituent models to enhance the robustness of noisy data, thereby diminishing noise’s impact on decision-making. In conclusion, the strategic utilization of ensemble models in our study aims to enhance predictive accuracy and robustness, underscoring their effectiveness in improving predictive modeling outcomes.

In this research, we utilized the two-step mean-weighted technique offered by the SDM package in R software to create a model. Initially, predictions were generated for RF, GBM, SVM, and GLM models through multiple iterations (cross-validation). Subsequently, the average of the predictions within each model was calculated. These averaged predictions were then combined with a weighted average (based on validation results) to determine the final values of the ensemble model. To develop an effective model for wind erosion monitoring, remote sensing indices were computed, and information regarding the eroded areas was extracted as inputs for the model. The modeling process was conducted for the years 2008 and 2023. An ensemble model was employed to monitor wind erosion, leveraging the collective impact of the models created in 2008 and the remote sensing data from 2023 to predict wind erosion. The resulting wind erosion map for 2023 will serve as a means to validate the model’s predictions. Subsequently, the study will evaluate the trends in wind erosion changes during the 15-year statistical period.

**Fig 2:**
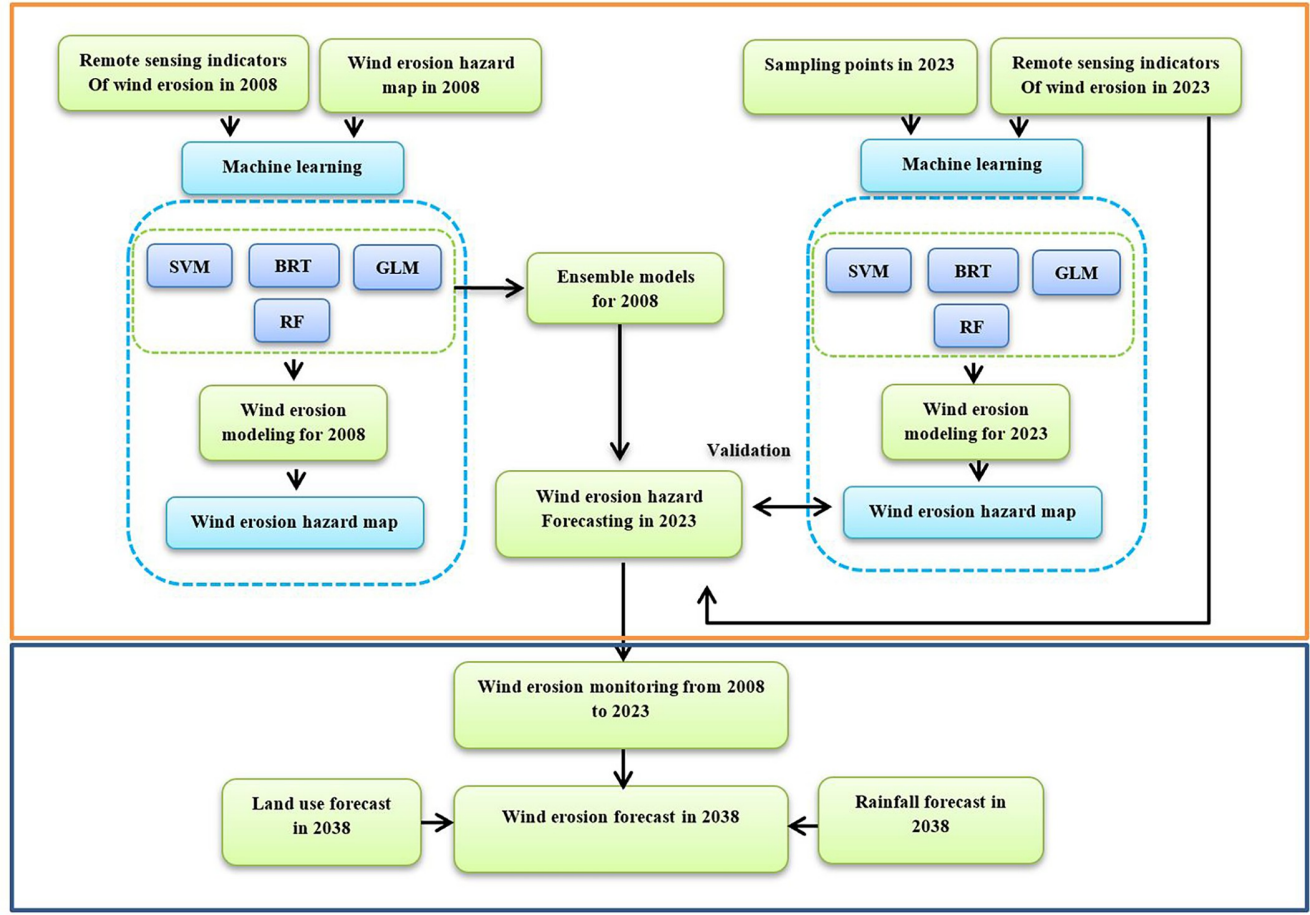
Methodological flowchart for this study, generated in Microsoft Office Word 2023 (https://www.microsof.com).

To predict wind erosion in 2038, taking into account the constant conditions, two scenarios of land use change and precipitation were used. Future land use change was obtained using the Markov chain model and precipitation using the sixth report of the Intergovernmental Panel on Climate Change (IPCC). The Markov chain model works as a random process in which the future state of a pixel depends only on its previous state and is predicted based on it [30] To predict land use changes in 2038, maps of 2023 and 2013 were used in Trust software using Markov chain model. The GCM models are the best available tools for climate modeling [31–34]. Therefore, among the various large-scale models in the sixth report of the IPCC, CanESM2 model, RCP2.6, RCP4.5, and RCP8.5 scenarios were used in this study. The framework of the present study is shown in Fig 2. Moreover, no permits were required for the work conducted in this study as it did not involve any activities that required authorization from any specific authority. No permits were required for using the software because it falls under fair use for personal or educational purposes, and does not involve any commercial use or distribution. We have carefully listed and cited all software used in this research.

## 3. Results

### 3.1 Selection of remote sensing indices for wind erosion modeling

To model wind erosion using remote sensing, a total of 8 indicators were selected based on a comprehensive review of relevant sources (Table 1). These indicators include surface texture, soil moisture, soil salinity, wind speed, percentage of vegetation cover, land use change, DEM, and rainfall. To evaluate the surface texture of the soil, the tab grain soil index (TGSI) was chosen, as it indicates changes in the size of surface soil grains, which is an important sign of soil degradation and wind erosion monitoring. For assessing soil salinity, the NDSI, VSSI, and SI indices were employed. Among them, the NDSI index showed a high Pearson’s correlation coefficient (0.88), low absolute average prediction errors RMSE (8.54), and an appropriate detection coefficient (R2 = 0.59), making it suitable for modeling purposes.

**Table 1:** The selection of indicators based on their correlation with ground surface data.

Indicators	Remote sensing indicators	Ground surface data	Correlation rate between satellite indicators and ground surface data			Reference
			R^2^	RMSE	Pearson coefficien	
Soil texture	TGSI	Soil surface profile	0.76	0.68	0.79	[35, 36]
EC	NDSI		0.67	6.34	0.82	
Rainfall	Chrips	Synoptic stations	0.55	134.23	0.77	[37]
Vegetation percent	NDVI	Vegetation percentage in plots	0.81	1.26	0.83	[38]
Management	Land use changes	Map of ground evidence	0.71	0.64	0.87	[39, 40]

For precipitation evaluation, the Chrips satellite data was found to be suitable based on correlation criteria, after examining data from TRMM GPM and PERSIANN satellites. Thus, the Chrips satellite data was utilized to assess the amount of precipitation in the region. The utilization of monthly satellite data from Chirps offers a substantial advantage in precipitation modeling. Chirps provide a 40-year dataset of rainfall, facilitating modeling endeavors and offering user-friendly accessibility. While some studies have indicated limitations in Chirps’ accuracy for daily precipitation estimation, particularly at finer temporal scales, it demonstrates superior performance at monthly scales. Therefore, this study opted to utilize monthly Chirps satellite data to address these concerns and ensure robustness in precipitation estimation.

To evaluate vegetation cover, five indices were considered, including SAVI, EVI, AVI, DVI, and NDVI. Among them, the NDVI index exhibited the highest correlation with ground data, specifically the vegetation percentage in the plots, making it the chosen index for wind erosion modeling. factors, an important aspect in wind erosion evaluation, were considered through the use of a land use change map as a remote sensing index. The validation of this map was conducted using ground evidence collected during field visits and Google Earth images.

The wind speed map was generated using data obtained from an anemometer station and interpolation techniques. The DEM for the area was obtained from the USGS website. For assessing soil surface moisture, the NDMI and MI indices were employed. The NDMI index exhibited a strong correlation with ground data, leading to its selection for wind erosion modeling purposes.

### 3.2 Wind erosion modeling

**Fig 3:**
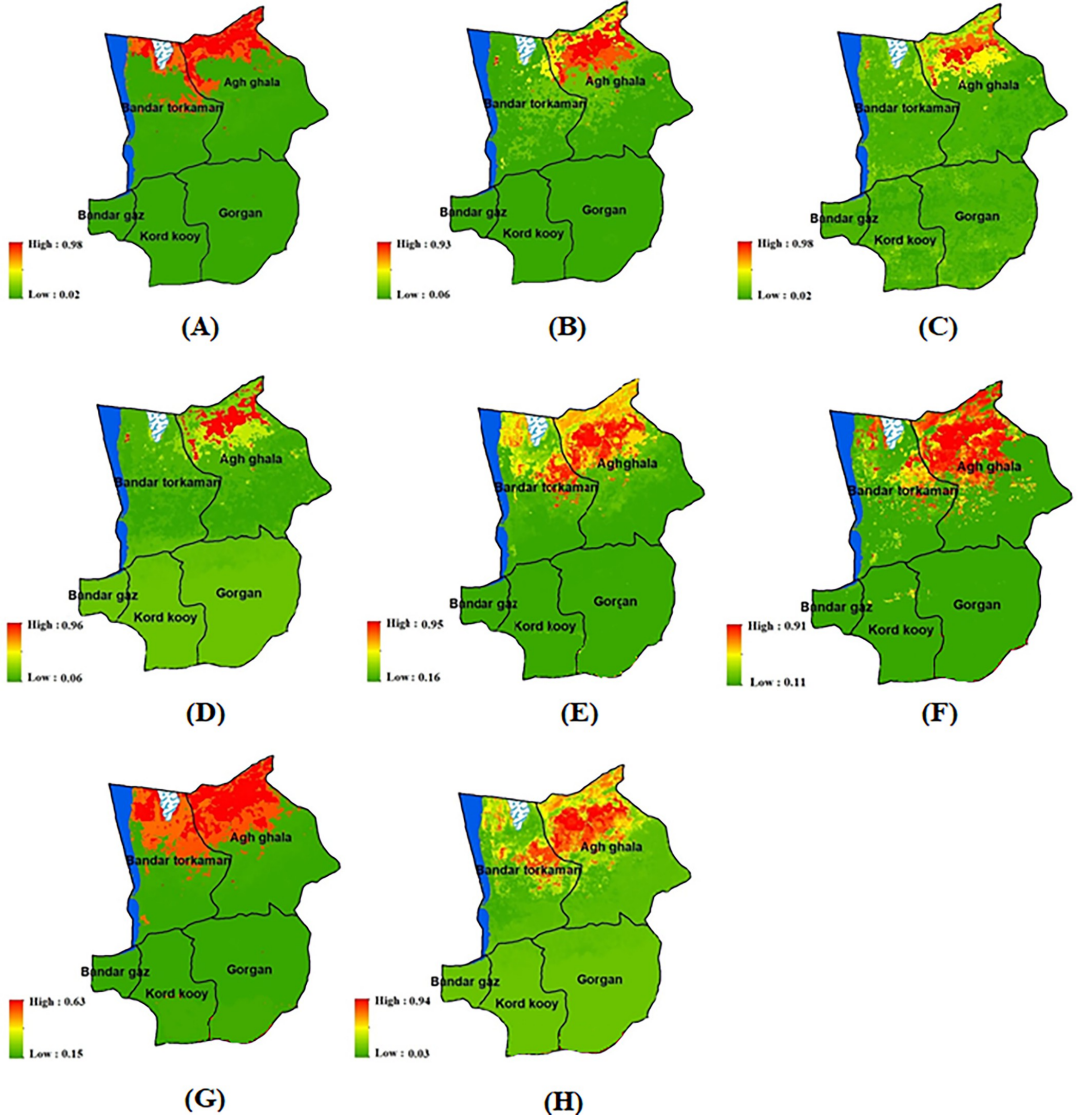
The maps illustrating wind erosion in northeastern Iran for 2008 and 2023, generated using various models from the SDM package. In 2008, the GBM, GLM, RF, and SVM were represented by labels A-D respectively. Similarly, in 2023, the GBM, GLM, RF, and SVM are denoted by labels E-H respectively, generated in the ArcGIS 10.7.1 software (www.esri.com) and R 4.3.2 software (www.r-project.org).

**Fig 4:**
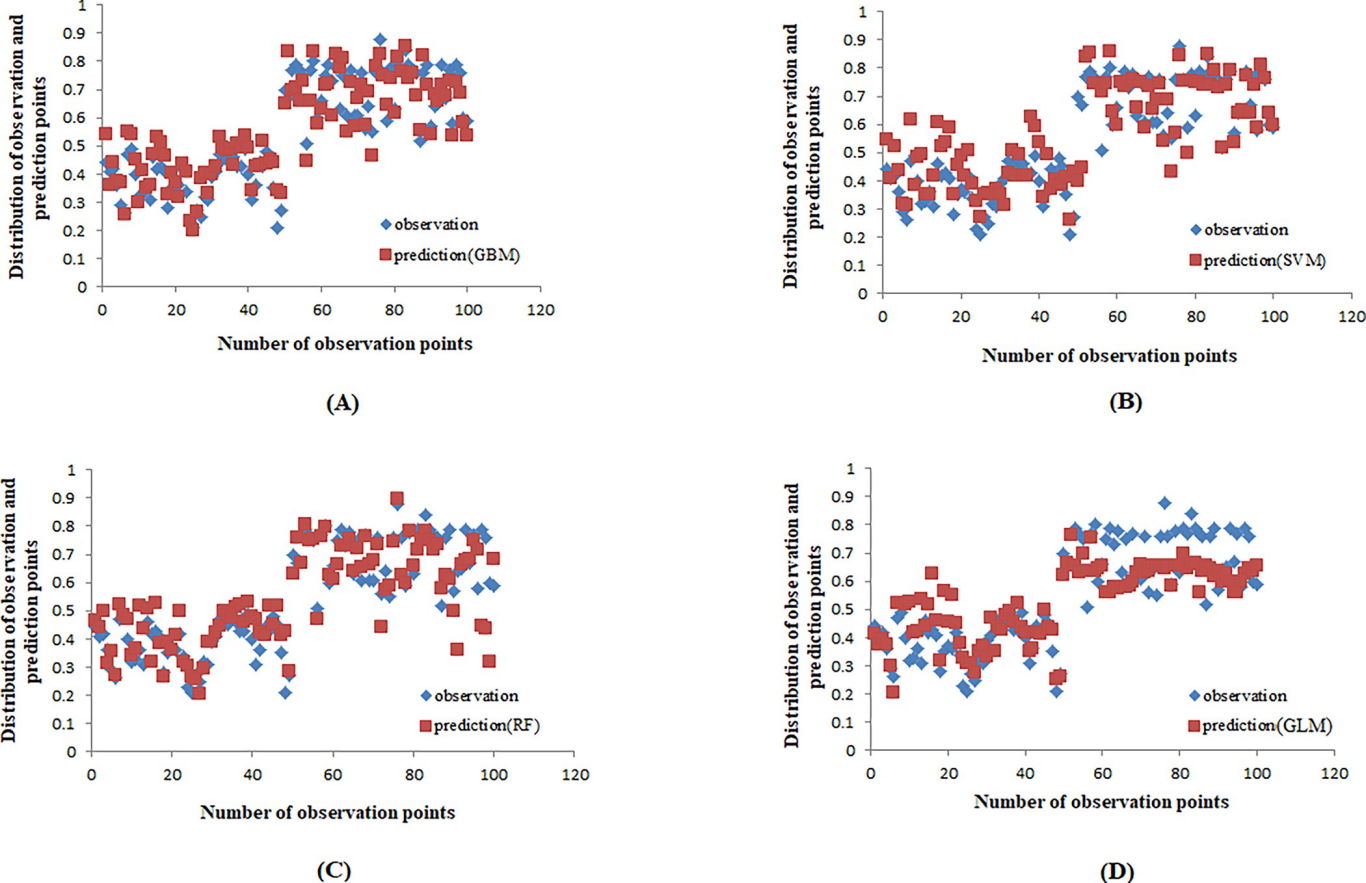
Comparing the performance of each machine learning method with observational data, A) GBM, B) SVM, C) RF, and D) GLM.

After generating maps displaying remote sensing indicators and identifying the presence and absence of wind erosion, the modeling process was initiated (S2 Data). The wind erosion modeling was conducted for the years of 2008 and 2023 using the sdm package within the R software environment (Fig 3). Except for the GLM, all three models exhibited strong predictions of wind erosion in the northwest region (north of Agh Qala city) in 2008. Additionally, the results indicated a progression of wind erosion risk from the northwestern areas in 2008 towards the northern and central regions of the study area by 2023. To evaluate the modeling results, statistical parameters such as the kappa coefficient, ROC, and TSS were employed (Table 2). Also, each machine learning method’s performance was evaluated against observational data from 2023. Findings indicated that the GBM model exhibited the strongest correlation (R = 0.94), while the GLM model had the weakest correlation (R = 0.83) when compared to the observational data (Fig 4).

**Table 2:** Performance evaluation of models based on various indicators in 2008 and 2023.

Methods	GLM		RF		GBM		SVM	
	2008	2023	2008	2023	2008	2023	2008	2023
AUC	0.87	0.82	0.92	0.86	0.88	0.95	0.90	0.81
TSS	0.65	0.73	0.82	0.63	0.57	0.79	0.74	0.69
KAPPA	0.77	0.89	0.96	0.84	0.61	0.95	0.80	0.91
Accuracy	0.78	0.80	0.85	0.82	0.80	0.87	0.81	0.82

Based on the modeling conducted in 2008, the RF model (AUC = 0.92, TSS = 0.82, Accuracy = 0.85, and kappa = 0.96) and the GBM model (AUC = 0.95, TSS = 0.79, Accuracy = 0.87 and kappa = 0.95) demonstrated superior performance in 2023. These results indicate the presence of uncertainty in the model outcomes. Therefore, to effectively monitor and investigate changes in wind erosion, ensemble models should be utilized.

### 3.3 Wind erosion monitoring using an ensemble model

For this research, we employed an ensemble model that integrated wind erosion data from 2008 with remote sensing indices from 2023 to forecast wind erosion in the future. In this study, to produce a set model, the weight of each model was determined based on its performance. The performance of the models was evaluated using AUC, TSS, and KAPPA criteria. The weights were calculated and normalized based on the average of these criteria. The final weights of RF, GBM, SVM, and GLM models were 0.286, 0.215, 0.257 and 0.241, respectively. These weights were used in a two-stage weighted averaging process to produce the final prediction of the ensemble model. This method has increased the prediction accuracy and reduced the variance by allocating more weight to the models with better performance.

**Fig 5:**
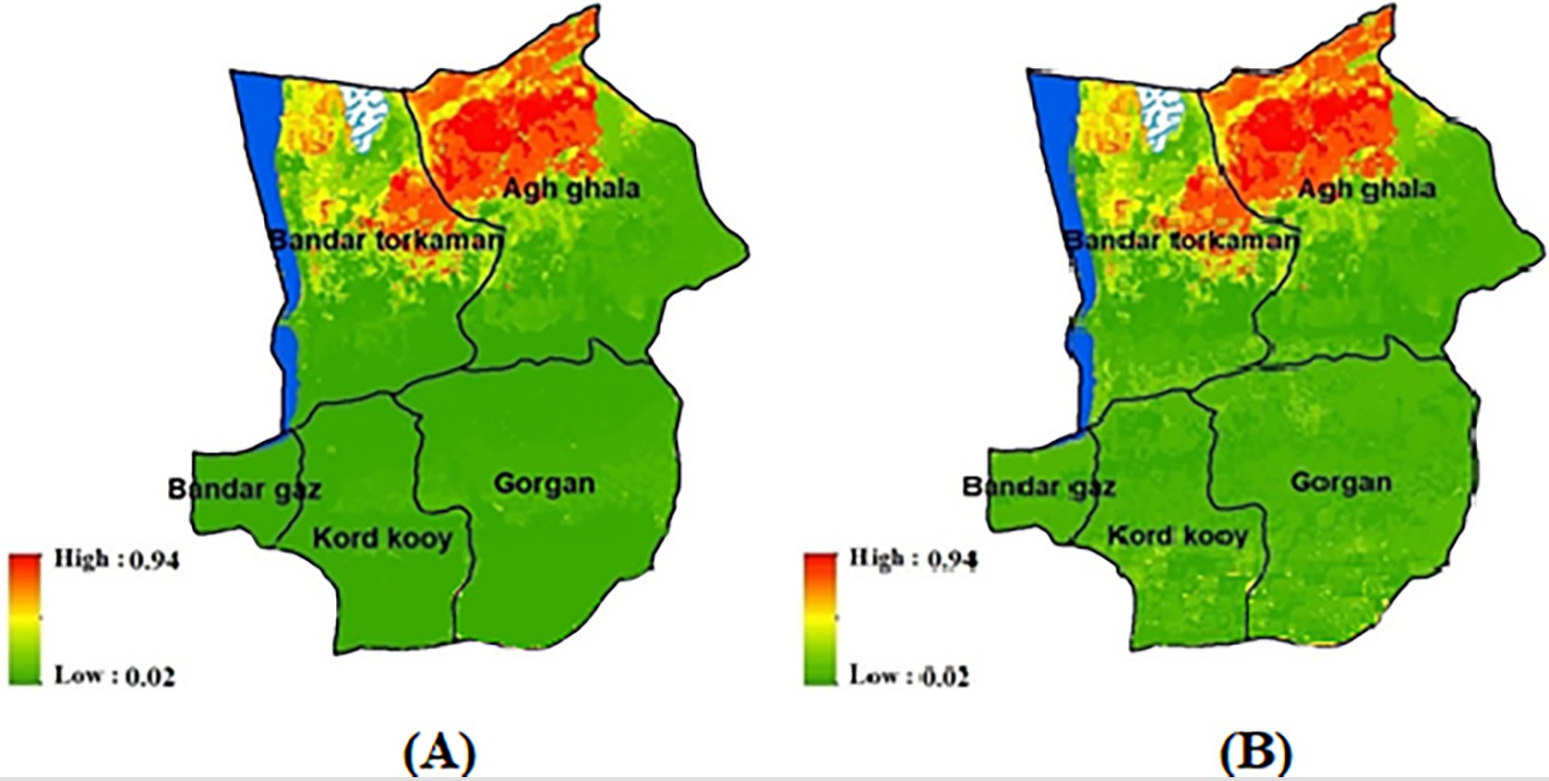
A) Wind Erosion Forecast map in 2023. B) Wind Erosion Map in 2023 using the ensemble model, generated in the ArcGIS 10.7.1 software (www.esri.com) and R 4.3.2 software (www.r-project.org).

The prediction generated by the ensemble model for wind erosion in the year 2023 accurately identified the affected regions in the northeast and sporadically in the southern parts of the studied area (Fig 5). To validate the performance of the ensemble model, we compared its predictions with the wind erosion map of 2023. The results of the validation indices (AUC = 0.92, TSS = 0.87, and kappa = 0.93) demonstrate the high accuracy of the forecast produced by the ensemble model. To ensure the performance of the ensemble model, the performance of the model was evaluated over two years using statistical parameters (Table 3). Based on the obtained results, the high correlation of 0.9 in most statistical indicators means that the ensemble model has an excellent performance. Also, the results of the significance level show that there is no significant difference between the results of the model and the real data (Table 3).

**Table 3:** Performance evaluation of ensemble model based on various indicators in 2008 and 2023.

Method	Ensemble model		Approximate significant	
	2008	2023	2008	2023
AUC	0.91	0.92	0.09	0.03
TSS	0.85	0.87	0.31	0.06
KAPPA	0.95	0.93	0.1	0.01

**Fig 6:**
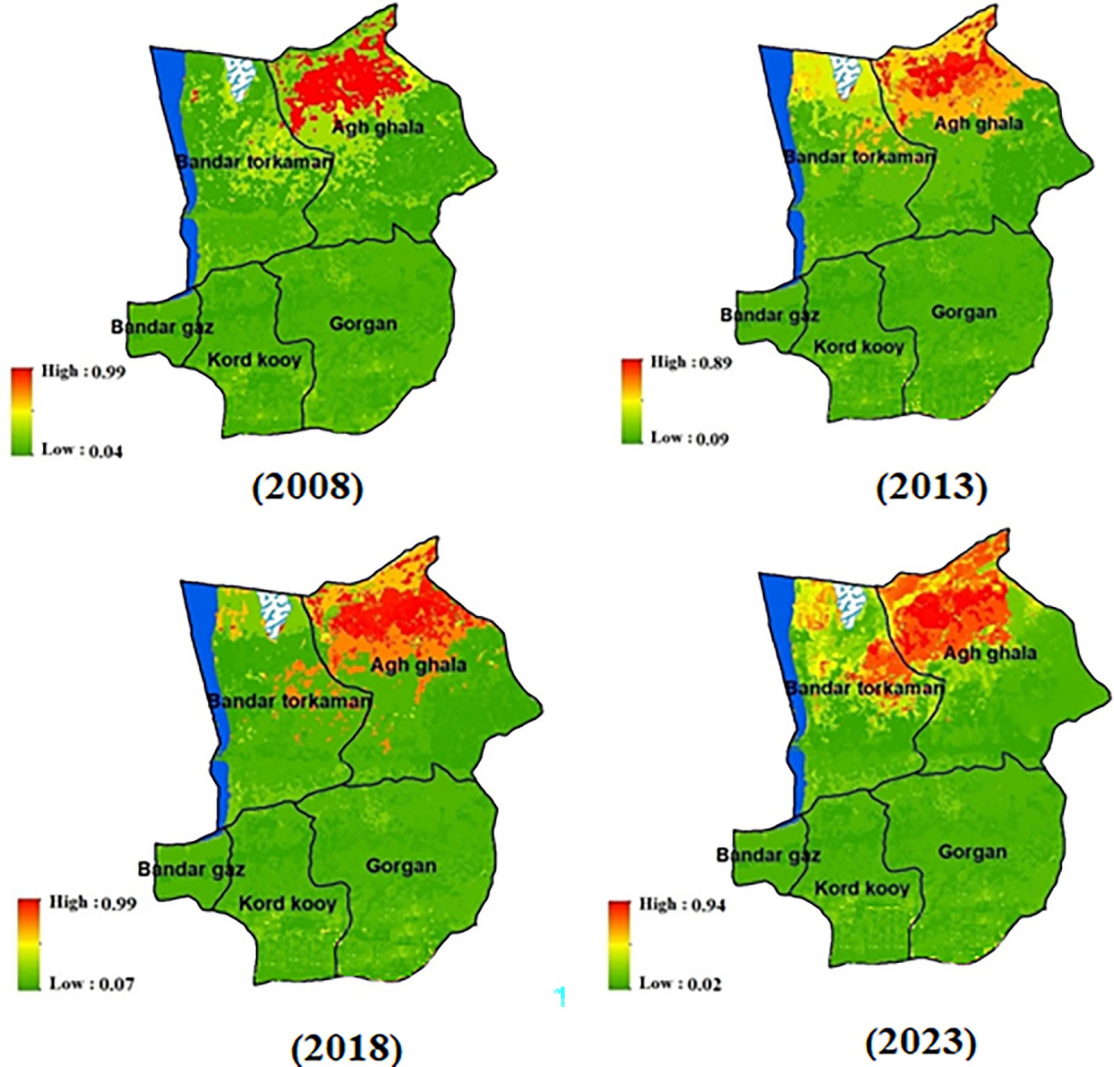
Wind erosion monitoring during the 15 years (2008–2023) using the ensemble model, generated in the ArcGIS 10.7.1 software (www.esri.com) and R 4.3.2 software (www.r-project.org).

Once we ensured the reliability of the model’s prediction, we proceeded with monitoring wind erosion from 2008 to 2023 using the ensemble model (Fig 6). The area categorized as having low wind erosion has expanded by 4,373 hectares, encompassing a total of 230,653 hectares, compared to the previous coverage of 226,279 hectares. Conversely, the average wind erosion class has decreased by 16,131 hectares, reducing its extent from 150,987 hectares to 134,856 hectares. In contrast, the high wind erosion class has increased by 11,757 hectares, covering a total of 144,492 hectares, in comparison to the previous 132,734 hectares. Spatial analysis of the changes in wind erosion classes revealed that the majority of these alterations occurred in the northern regions, including areas surrounding the Sangartape desert, western regions, and near the Gomishan wetlands, along with the rangelands of Agh Qala and Gomishan cities. These regions have been identified as particularly vulnerable to wind erosion, as illustrated in Fig 6.

### 3.4 The most important variables

**Fig 7:**
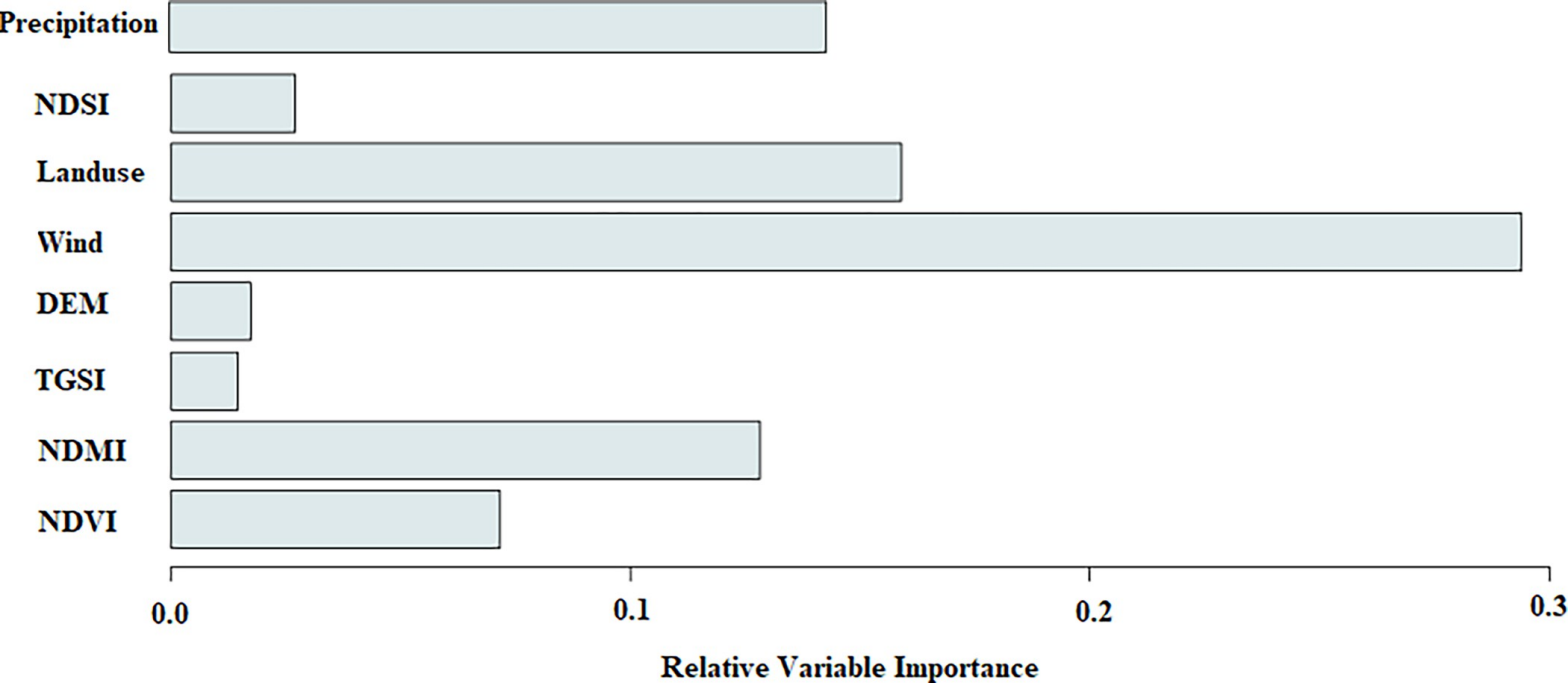
Importance of variables in the study area in 2023, generated in the R 4.3.2 software (www.r-project.org) and Microsoft Office Excel 2023 (https://www.microsof.com).

Gaining a comprehensive understanding of the contributing factors to wind erosion holds immense significance in the realm of environmental research, conservation of natural resources, and the pursuit of sustainable development. By discerning these variables, we can effectively implement strategies aimed at mitigating wind erosion, improving soil management practices, and fostering responsible land utilization. Determining variable importance in our analysis was conducted using the SDM package within the R software environment. This method calculates the relative importance of each variable based on its contribution to the model’s predictive performance. Consequently, the significance of the examined indicators within the superior model (GBM) was determined in 2023. The vertical axis of graph 7 shows the variables selected for modeling and the horizontal axis shows the importance of each of these variables in the modeling process. The findings revealed that wind speed, land use, rainfall, NDMI, and NDVI held greater importance compared to NDSI, TGSI, and DEM, respectively (Fig 7). This knowledge assists in making well-informed decisions for effective management and planning, thereby minimizing the associated risks linked to wind erosion.

### 3.5 Prediction of future wind erosion

**Fig 8:**
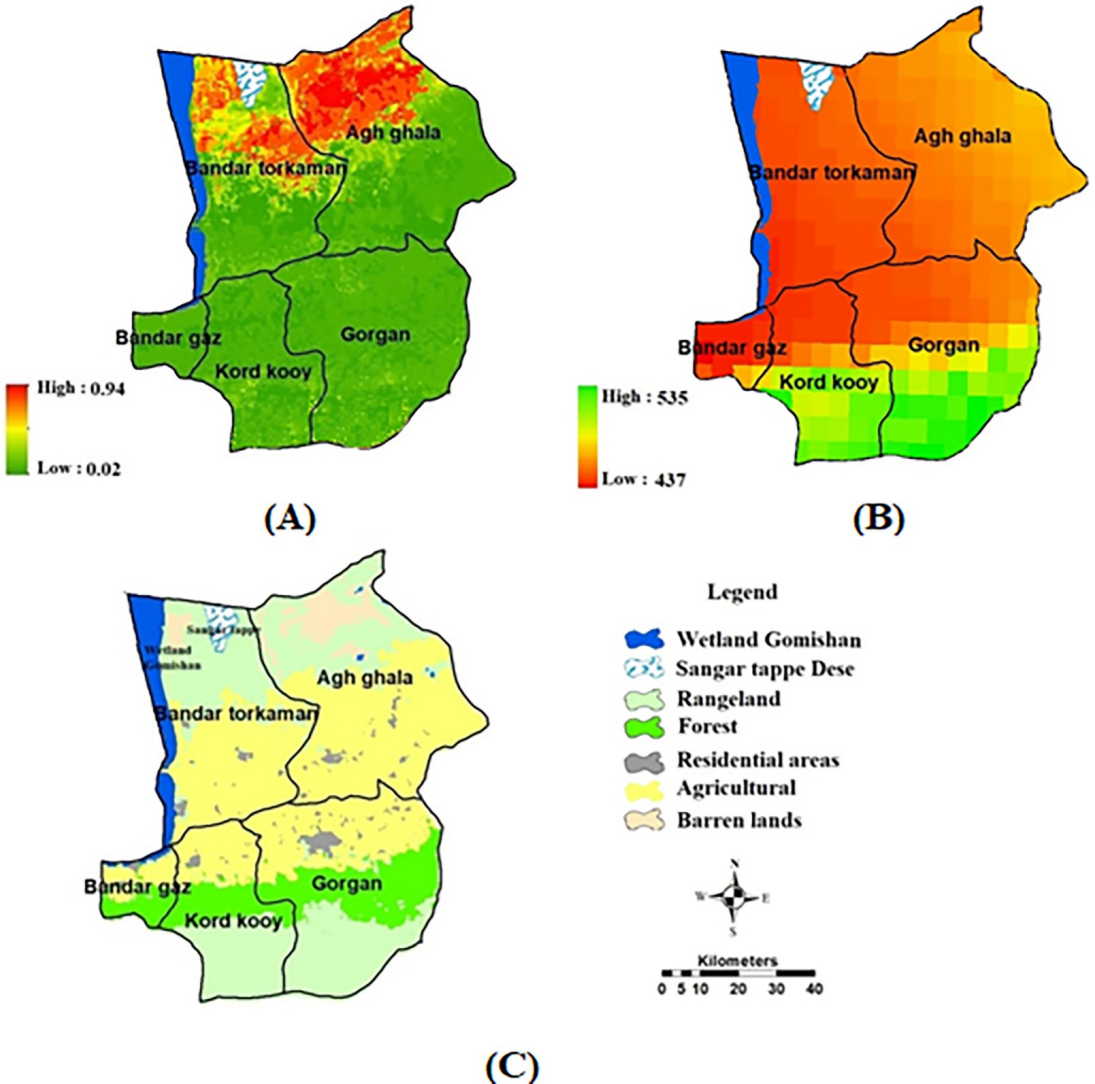
A) Forecast map of wind erosion in 2038; B) land use scenario in 2038; C) Rainfall scenario of 2038, generated in the ArcGIS 10.7.1 software (www.esri.com), R 4.3.2 software (www.r-project.org), and TerrSet 2020 software (https://clarklabs.org).

To predict wind erosion in the year 2038, we conducted simulations considering changes in rainfall and land use, assuming other conditions remain unchanged. The results of the rainfall simulations for 2038 indicate a decrease of 47 mm in rainfall at Gorgan station, while there is an increase of 34 mm, 18 mm, 21 mm, and 15 mm respectively in the cities of Bandar Turkmen, Kordkoi, Agh Qala, and Bandargaz. Based on the average simulated precipitation for the scenarios, a precipitation map for 2038 was created (Fig 8).

In terms of land use prediction for 2038, the Markov chain model revealed an increase of 8246 hectares and a decrease of 2589 hectares in the utilization of barren lands and low-density rangelands. These changes are expected to significantly contribute to the increase in wind erosion in the region (Table 4). Assuming the surface texture, soil moisture and salinity indicators, wind speed, percentage of vegetation and DEM remain constant, along with the projected rainfall and land use for 2038, wind erosion was predicted using an ensemble model (Fig 8). According to the predictions, wind erosion is anticipated to rise by 23% (equivalent to 2704 hectares) in 2038, with erosion spreading from the north to the central areas of the study.

**Table 4:** Investigating the trend of land use class changes.

Class	Land use	2023 (hectares)	2038 (hectares)	Percent changes
**1**	Water	20019.58	15119	-0.8
**2**	Forest	61189	51982	-1.51
**3**	Low density rangelands	165588	168117	0.42
**4**	Low density forest	16715	9543	-1.17
**5**	Residential	27840	71760	7.2
**6**	Agricultural	274934	241234	-5.53
**7**	Barren lands	43158	51405	1.35
**Total**		609446	609446	

## 4. Discussion

### 4.1 Wind erosion modeling and monitoring

The modeling involved determining whether wind erosion was present or absent in the region for 2008 and 2023 [37]. After carefully examining various sources, eight remote sensing indicators were chosen for analysis. Wind erosion modeling for 2008 and 2023 was carried out using four different methods from the SDM package in the R program. The findings showed that the RF model yielded the most accurate results in 2008, while the GBM model performed better in 2023. The RF model’s effectiveness can be attributed to its capability to continuously adjust and replace important factors influencing the desired outcome. This results in the creation of multiple decision trees that are used for prediction, as explained by Breiman (2001) [41]. However, it is important to note that this algorithm is comparatively more complex and time-consuming than other machine learning techniques, as mentioned by Terrados-Cristos et al. (2023) [42]. On the other hand, the GBM model delivers acceptable performance partly because of its self-evaluation and self-correction capabilities during the construction of multiple trees. This model also exhibits high flexibility, allowing it to effectively ensemble complex and diverse datasets to obtain the final result, as discussed by Biau and Scornet (2016) [43]. Nonetheless, the construction of multiple trees with dense foliage (controlled by the depth parameter) makes the model more susceptible to overfitting, where it may become overly sensitive to the training data.

The variations observed in the outcomes of the models indicate a certain level of uncertainty among them. Consequently, it has become necessary to employ an ensemble approach to address this issue [38]. To mitigate the uncertainty in predictions, utilizing a superior model is crucial, and employing a hybrid approach is considered an effective solution for reducing prediction uncertainty in modeling [19]. In this study, an ensemble modeling technique was employed to integrate the results of individual models, enhancing the accuracy of forecasting. Various methods exist for combining model predictions in the integrated model, and in this research, the average method was utilized. In the average method, the predictions from all models are aggregated by summing them, and the final outcome is obtained by averaging these predictions [13]. Given that different models may excel under different circumstances, combining these models can yield the optimal output and minimize uncertainty [44].

Moreover, the ensemble model of 2008 and remote sensing indicators of 2023 were used to monitor wind erosion. After confirming the correctness of the predicted wind erosion map of 2023, wind erosion monitoring was done for 15 years. The analysis of wind erosion spatial changes reveals an intensification of erosion in several regions. These include the northern areas near the Sangartape desert, the northeastern abandoned lands, the western margins of wetlands, and the central regions encompassing marshlands, alluvial sediments, and the rain fields of Agh Qala in Gomishan.

The abandoned and barren lands are particularly susceptible to wind erosion due to factors such as the absence of a compacted soil surface layer, limited moisture, and insufficient vegetation. The mismanagement and land use practices in these areas have led to the formation of an activated Nebka facies, indicating an increase in the particles undergoing wind erosion in this region. Zhou, et al (2008) [45] emphasized in their study that the absence of vegetation and the presence of pits are clear indications of the intensity of sediment removal and the escalation of wind erosion in the area. Furthermore, the soil surface in these regions is characterized by dryness and a lack of adhesion, primarily due to inadequate humidity and insufficient land management practices. This leads to increased activity and movement of the soil particles [39]. In their research, Akbari et al. (2020) [46] also highlighted that visible sign of erosion on the earth’s surface, such as sandy areas, hills, and windswept lands, serve as warning signals for wind erosion. These signs indicate the lack of stability and sensitivity of the constituent particles to the wind regime and dust [39].

### 4.2 Identifying influential variables and predicting the future

This study has identified the primary factors influencing the strength of wind erosion, namely wind speed, land use, and precipitation. These factors have a notable impact on the intensity of wind erosion in the specified regions. Wind speed, in particular, plays a major role by causing the movement of soil particles on the surface. The prevailing west-to-east winds facilitate the transportation of sand from coastal lands to the plain lands in the east, indicating the transfer of sand from coastal salt plains to the hilly plains in the northern part of Golestan province [10]. Over time, the increase in population and subsequent demand for agricultural products has led to changes in land use patterns. These changes in land use have contributed to the intensity of wind erosion. Notably, the southern region of the research area serves as a significant center for dry wheat cultivation in Iran. It is evident that there has been a substantial expansion in the cultivated area in this region, which has grown tenfold compared to the past two decades. This expansion clearly indicates a significant shift in land use patterns in the area. In order to forecast wind erosion in the year 2038, the researchers utilized the insights gained from examining the influential variables. Two scenarios were taken into account: changes in land use and precipitation. The wind erosion map for 2038 vividly depicts the progression of wind erosion, starting from the northern regions and advancing towards the central regions. Based on the findings, if erosion control is neglected and effective solutions to reduce wind erosion are not implemented, there could be a 23% increase (equivalent to 2704 hectares) in the areas affected by wind erosion by 2038. The adoption of unsustainable agricultural practices, coupled with changes in land use, has the potential to contribute to desertification, which in turn can lead to a further escalation of wind erosion in the future. To address this issue, it is of utmost importance to implement solutions that prioritize the protection of ecological and water-sensitive areas, preservation of natural resources, promotion of sustainable agriculture, and optimization of land usage. These measures can play a crucial role in mitigating the impact of wind erosion.

## 5. Conclusion

Wind erosion and its management have consistently been important topics of concern worldwide. This phenomenon has a direct impact on critical environmental components such as soil, water, and air. Consequently, the modeling, monitoring, and prediction of wind erosion play a crucial role in environmental studies. In this particular study, the researchers employed four models using the SDM statistical package, and the evaluation of accuracy results allowed for the identification of the most effective model. The RF method is strong against over fitting and can handle large datasets with high dimensionality, but its interpretability is sometimes limited due to its complex structure. On the other hand, GLM method is simpler and assumes a linear relationship between predictors and the response variable, which may restrict their accuracy in capturing complex, non-linear patterns in environmental data. The GBM is powerful and flexible but can over fit, particularly with noisy data. The SVM performs well in high-dimensional spaces but may struggle with very large datasets due to computational costs. Comparing the performance of an ensemble model with individual models using statistical methods, the ensemble model with the normalized average of Kappa, TSS, and AUC criteria in 2008 and 2023 outperformed the individual models. The results at a 95% significance level indicate the positive and significant impact of using an ensemble model in wind erosion monitoring. This research used an ensemble model with a unique approach to monitor wind erosion, enhancing predictive performance by combining strengths of individual models, reducing over fitting risks, and improving generalizability across different spatial and temporal scales. This approach allows for the inclusion of new data and adaptation of the model to similar environmental conditions in other regions, making it a versatile tool for wind erosion monitoring.

The variations observed in the results among the different models highlight the inherent uncertainties associated with modeling wind erosion. Therefore, adopting an ensemble approach proves to be a suitable solution for minimizing these uncertainties. The monitoring results of wind erosion, obtained by implementing the ensemble model, reveal an increase in erosion intensity in areas surrounding the Sangartape desert, abandoned lands, wetland margins, salt marshlands, alluvial sediments, and rain fields in Agh Qala and Gomishan cities. Based on these findings, appropriate measures can be implemented to address and mitigate the impact of wind erosion in these specific locations. Based on these findings, it is recommended to employ adaptive and biological measures as strategies for addressing the challenges posed by wind erosion. These measures include promoting the growth of plant communities that can withstand soil and water salinity, increasing the presence of plants resistant to salinity and drought in degraded rangelands, adopting conservative agricultural practices that emphasize the preservation of crop residues, and establishing windbreaks along the edges of cultivated land. For dried wetland water ecosystems, sustainable measures to prevent dust spread and wind erosion involve securing water rights, maintaining moisture on surfaces, and preserving plant species that can tolerate salt. While there may have been delays and uncertainties in achieving desired outcomes, it is imperative to urgently implement a comprehensive plan to tackle the issues of dust and wind erosion in Golestan province. This urgency arises from the escalating occurrence of dust events in recent years and the irreversible consequences associated with them. By taking prompt action based on these recommendations, effective mitigation and management strategies can be implemented to combat dust and wind erosion in the region.

## Supporting information

S1 Data Field-based data for spatio-temporal wind erosion risk analysis. (XLSX)

S2 Data Spatio-temporal data mining modeling for wind erosion risk assessment. (XLSX)
